# Perspectives on the changing healthcare system: teaching systems-based practice to medical residents

**DOI:** 10.3402/meo.v18i0.20746

**Published:** 2013-09-02

**Authors:** Johanna Martinez, Erica Phillips, Oliver Fein

**Affiliations:** Weill Medical College, Cornell University, New York, NY, USA

**Keywords:** curriculum development, graduate medical education, core competencies, systems-based practice, healthcare systems

## Abstract

**Purpose:**

The Accreditation Council for Graduate Medical Education restructured its accreditation system to be based on educational outcomes in six core competencies. Systems-based practice is one of the six core competencies. The purpose of this report is to describe Weill Cornell Medical College's Internal Medicine Residency program curriculum for systems-based practice (SBP) and its evaluation process.

**Methods:**

To examine potential outcomes of the POCHS curriculum, an evaluation was conducted, examining participants': (1) knowledge gain; (2) course ratings; and (3) qualitative feedback.

**Results:**

On average, there was a 19 percentage point increase in knowledge test scores for all three cohorts. The course was rated overall highly, receiving an average of 4.6 on a 1–5 scale. Lastly, the qualitative comments supported that the material is needed and valued.

**Conclusion:**

The course, entitled Perspectives on the Changing Healthcare System (POCHS) and its evaluation process support that systems-based practice is crucial to residency education. The course is designed not only to educate residents about the current health care system but also to enable them to think critically about the risk and benefits of the changes. POCHS provides a framework for teaching and assessing this competency and can serve as a template for other residency programs looking to create or restructure their SBP curriculum.

## Introduction

Within the ever-changing US healthcare system, it is inevitable that Graduate Medical Education (GME) must also continuously transform itself. This notion has been acknowledged nationally by organizations such as the Accreditation Council for Graduate Medical Education (ACGME) and the Institute of Medicine (IOM). Indeed, the IOM's landmark publication ‘To Err is Human: Building a Safer Health System and Crossing the Quality Chasm: A New Health System for the 21st Century’ has highlighted that ‘fundamental changes are needed in the organization and delivery of health care’ (1). The ACGME has responded to this call with the development of the Next Accreditation System (NAS) – which aims to: (1) enhance the ability of the peer-review system to prepare physicians for practice in the 21st century; (2) accelerate the ACGME's movement toward accreditation on the basis of educational outcomes; and (3) reduce the burden associated with the current structure and process-based approach (2, 3). The educational outcomes are grounded in the six core competencies of patient care, medical knowledge, practice-based learning and improvement, systems-based practice (SBP), professionalism and interpersonal skills and communication.

These mandated changes in residency education are timely and even more important in the era of healthcare reform. In March 2010, the Patient Protection and Affordable Care Act (P-PACA) was signed into law, and in June 2012 it was declared constitutional by the US Supreme Court. P-PACA focuses on expanding medical coverage, controlling healthcare costs, improving the healthcare delivery system and more tightly regulating private health insurance. It does so by mandating that every US citizen have health insurance and by eliminating pre-existing condition exclusions and annual and lifetime limits from private health insurance. It creates state-based *health insurance exchanges* for individuals and small group markets (4)—changes that will have a significant impact on every practicing physician in the United States. Unfortunately, many residency programs still fail to recognize the divide which persists between what is currently taught in the core competency of SBP and the demands of a changing healthcare system (5, 6). It is therefore crucial to provide a structured curriculum that recognizes and addresses these issues in GME.

SBP is the ability to ‘demonstrate an awareness of and responsiveness to the larger system of health care and the ability to effectively call on system resources to provide care that is of optimal value’ (7). Specifically, residents must be able to: (1) work effectively in various healthcare delivery settings and systems relevant to their clinical specialty; (2) coordinate care within the healthcare system; (3) incorporate considerations of cost awareness and risk benefit analysis in patient or population-based care, as appropriate; (4) advocate for quality patient care and optimal patient care systems; (5) work in interprofessional teams to enhance patient safety and improve patient care quality; and (6) participate in identifying system errors and implementing potential solutions (7).

Comparatively speaking, SBP is arguably less evident than patient care, medical knowledge, professionalism, interpersonal skills and communication – making it more challenging to teach and assess (8–10). Despite these challenges, educators, from the time of managed care reform, ‘have widely acknowledged the need to promote competencies essential to managed care practice’ (3, 11). Moreover, residents, too, have indicated feeling ‘unprepared to work in a managed care practice’ – reflecting how future practices may look under the current healthcare reform proposals (3, 11). In response to this gap in SBP among graduate medical trainees, we developed the Perspectives on the Changing Healthcare System (POCHS) curriculum.

The primary objective of the POCHS curriculum is to increase residents’ knowledge around SBP including: (1) specifics of healthcare organization and delivery; (2) issues related to cost, access, and quality; and (3) the proposed changes to the US healthcare system. From a programmatic standpoint, supplemental efforts focus on: (1) obtaining feedback on the curriculum – from delivery methods to quality of content and (2) eliciting qualitative feedback on the perceived benefits, and how to better teach core content in a novel, effective manner.

## Methods

### Setting and participants

The curriculum was first developed as an educational partnership between managed care organizations and academic medicine (12, 13). A similar course was offered to medical students (14), but restructured for residents in 2007 to keep pace with the demands of the changing healthcare system. This report will focus on the current course, examining the experiences of three cohorts of PGY-3 residents at Weill Cornell Medical College as they complete their Internal Medicine training at three clinical sites: New York Presbyterian, Weill Cornell Medical Center, and Memorial Sloan Kettering Cancer Center and Hospital for Special Surgery.

The revised curriculum transitioned the delivery of material from didactic to more interactive sessions, and the content was geared more toward current trends in healthcare reform. Finally, the course evaluation process was revamped to better assess associated knowledge gain and elicit participants’ perceptions of its benefit.

The POCHS curriculum is a mandatory, one-week rotation in the PGY-3 Internal Medicine graduate curriculum, during which direct patient care is limited to one 3-hour evening practice session. The course is offered five times annually, resulting in a small group teaching environment with 8–12 participants per session. A content expert facilitates most sessions. Between October 2008 and January 2011, 142 participants completed this course. Cohorts were comprised predominantly (96%) of Internal Medical residents with some representation by other healthcare trainees (surgery residents, geriatric fellows and Master of Public Health students). The Weill Cornell Medical College Institutional Board approved this study.

### Program description

The four main components of the curriculum are: (1) Finance of the Healthcare System; (2) Organization of Medical Practice; (3) Healthcare Policy, Reform and Advocacy; and (4) Quality in Healthcare. The topics reviewed in each respective component are detailed below. Each component's learning objective is listed in Table 1.

**Table 1:** POCHS categories

Topics	Lectures	Learning objective
Healthcare System Finance	Healthcare costs, financial incentives, insurance plans—Medicare, Medicaid, private plans, HSA	To list three major differences between Medicare and Medicaid
Organization of Medical Practices	PPO, HMOs, medical homes, ACOs, health information technology	To articulate three benefits and three potential risks of an ACO or medical home
Healthcare Policy, Reform and Advocacy	Reform in Washington, P-PACA, Special Interest Groups—PHARMA, Comparative International Health Systems	To recognize the role of patient advocacy in improving health outcomes
Quality in Healthcare	Measures-structural, process, outcomes Malpractice, conflicts of interest	To identify a patient outcome that is influenced by a systems-based practice process measure

#### Finance of the healthcare system

The POCHS curriculum begins with an abbreviated introduction into the history of health insurance in the United States, and it features an in-depth discussion of Medicaid and Medicare. Details on the origins of health insurance – including its structure, organization, and financing – are also reviewed, as are the impacts on beneficiaries. To better understand how payments are made, basic health economics is touched upon; similarly, CPT (Current Procedural Terminology) and ICD-9 (International Classification of Disease – 9th edition) codes are reviewed. Residents also learn about Medicare's Part D prescription drug benefits, as well as The Medicare Rights Center – a not-for-profit organization that helps patients select the best prescription drug benefit plan for their condition. Finally, the differences between Medicare and Medicaid are contrasted with private/commercial health insurance.

#### Organization of Medical Practice

To understand the intricacies of the US healthcare system, residents learn the differences among private practice, group practices, accountable care organizations (ACOs), and medical homes – including differences in reimbursement for health maintenance organizations (HMOs), preferred provider organizations (PPOs), and fee-for-service providers. Finally, the use of health information technology (e.g., electronic medical records) is discussed, and general comparisons are made with other international health delivery systems.

#### Healthcare Policy, Reform and Advocacy

Understandably, this component of the course is continually updated to reflect changes to the current Federal health policy. During US presidential elections, candidates’ positions are typically explored and, most recently, the P-PACA is reviewed in detail. Regarding conflicts of interest that may arise during physicians’ interactions with industry, residents are given practical steps on being involved in change, reform, and patient advocacy. When addressing health advocacy the topic of health disparities is incorporated.

**Table 2:** Mean lecture evaluation ratings

Topics	Mean (SD)
Finance of the Healthcare System	3.4 (0.1)
Organization of Medical Practices	4.3 (0.3)
Healthcare Policy, Reform and Advocacy	4.1 (0.2)
Quality in Healthcare	4.2 (0.3)
Overall Score	4.6 (0.1)

Rating scale: 1–5.

#### Quality in Healthcare

One of the institution's lawyers discusses with residents ways in which practice style and behaviors can improve quality care, and trainees are also instructed on risk-reduction techniques that can be incorporated into their practices. The basic concepts of Quality Improvement (QI) process measures, structural measures, and outcome measures are introduced, and participants perform a reflective exercise using Medicare's Hospital Compare (http://hospitalcompare.hhs.gov) website to assess how our institution compares to other New York City hospitals with regard to myocardial infarctions and pneumonia care.

In particular, there are three components of the POCHS curriculum that are fairly unique: (1) Site Visit; (2) Debate; and (3) Rogers Colloquium.

Previously, the site visit has included a health insurance company (e.g., Blue Cross-Blue Shield), where residents were able to get first-hand experience of how claims are processed and either denied or approved. More recently, trainees have visited a large multi-specialty group practice – exposing them to an emerging healthcare delivery system. Visiting a local legislator's office is another addition to this component.

The debates are structured around controversial, contemporary topics – with residents divided into ‘pro’ and ‘con’ teams, each of which is given a bibliography and printed articles to research and read. Residents then engage in a formal, public debate in front of peers and selected faculty with related expertise. Prior debate topic areas have included McCain vs. Obama, pay-for-performance (P4P), the public option, and cost/comparative effectiveness research.

Named for Dr. David Rogers, a graduate of the Cornell University Medical College and the first President of the Robert Wood Johnson Foundation, the Rogers Health Policy Colloquium is a weekly seminar featuring prominent healthcare leaders from throughout the country. Ensuing discussions focus on improving patient care and safety based on assessing gaps in physicians’ knowledge, competence and performance, and generally include issues in domestic health policy – such as access, disparities, reform, policy, ethics, and history. In addition, the colloquium includes international health policy topics (e.g., WHO Essential Drugs Program) and issues related to resource-rich and -poor countries.

## Results

### Program evaluation

To examine potential outcomes of the POCHS curriculum, a cursory evaluation was conducted, examining participants’: (1) knowledge gain; (2) course ratings; and (3) qualitative feedback.

#### Knowledge

**Fig. 1:**
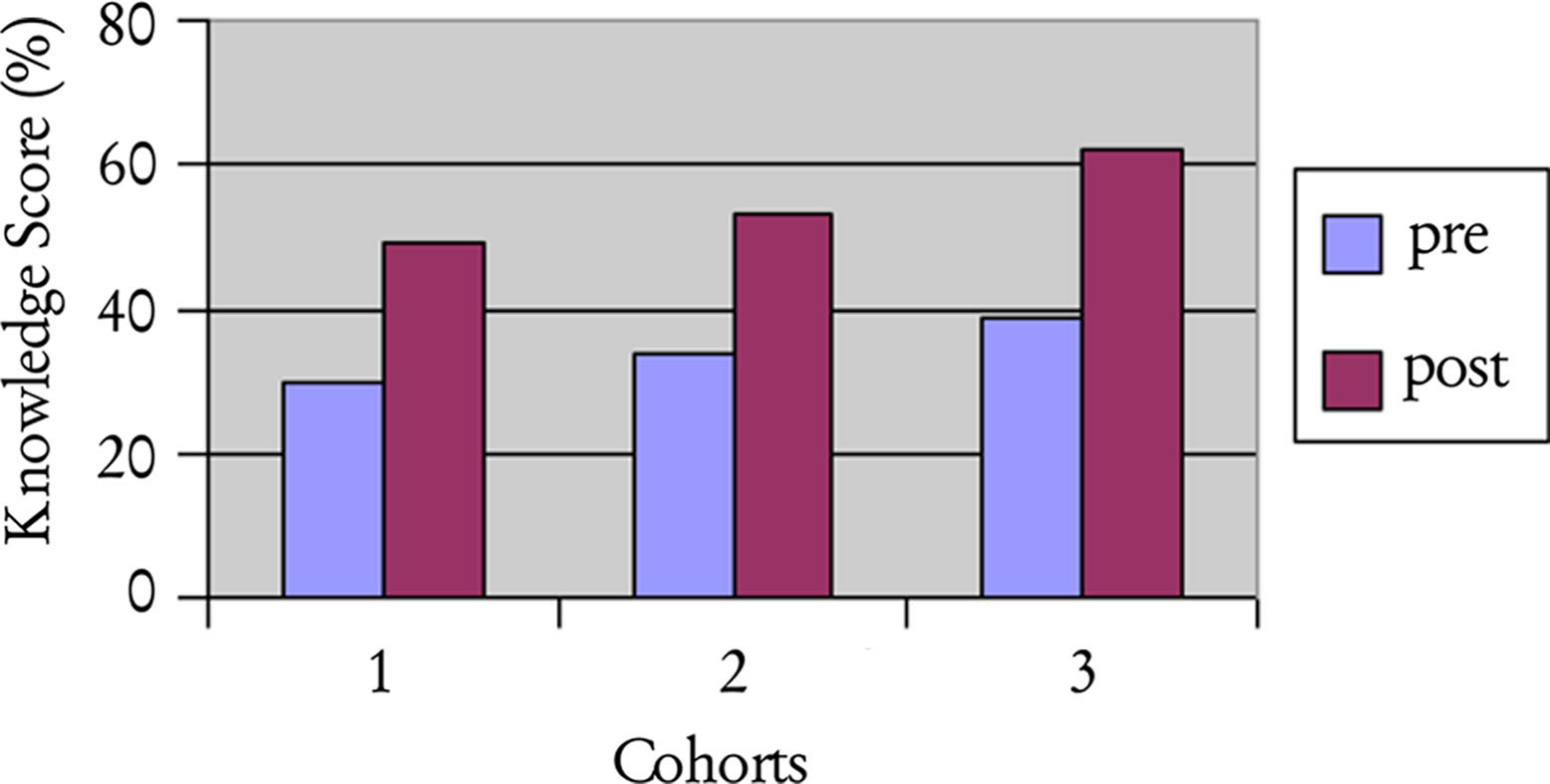
Knowledge score.

Derived from a 50-item multiple choice pre- and post-test, with 2 points awarded for each correct answer, a knowledge score was calculated for each resident. Internal consistency of the knowledge test was acceptable – with Cronbach's alpha (α) ranging from 0.69 to 0.77, with an average of 0.72. On average, there was a 19 percentage point increase in knowledge test scores for all three cohorts (*p*≤0.001), as well as a general trend toward higher scores in subsequent years (see Fig. 1). However, despite the increases in post-test scores, performance remains low (49–62%), which may be partly due to the lack of exposure and comfort with this material, as mentioned in the qualitative comments.

#### Course ratings

Participants also completed a written evaluation following each rotation, which included residents’ assessments of each instructor, satisfaction with the rotation, content delivery format, teaching venues, and bibliography. The average rating per lecture was 4.2 (SD 0.32) across all cohorts, and the overall course quality – which ranged on a 5-point Likert scale from ‘not appropriate for residency education’ to ‘one of the best residency rotations’ – averaged 4.6 (SD 0.08), see Table 2.

#### Qualitative feedback

During the final evaluation, residents are encouraged to freely respond to questions about content delivery and perceived benefits of the core material. In addition to transcribing their comments, time is allowed for open discussion. Comments centered around three basic themes: (1) relevance and importance of the material; (2) practical (clinical) impact; and (3) their involvement in healthcare reform. Representative comments included:

1. ‘Excellent course must be continued. We need more of this’.
2. ‘Essential and crucial information, one week not enough time’.
3. ‘The course must be included in [residency] curriculum, the only time we learn about healthcare’.

## Discussion

Based on evaluation data, the POCHS curriculum appeared to significantly enhance residents’ knowledge of SBP – responding to the ACGME mandate that residency programs provide evidence showing the degree to which the core competencies are attained (7). Their overall increased knowledge provides them with the tools to become ‘competent’ at systems-based practice. With ongoing changes in healthcare, it is crucial that GME afford residents a comparable curriculum in SBP. Based on our literature review of this area, more residency programs are, in fact, teaching SBP; however, a greater emphasis on related knowledge and skills is still warranted (6).

Most residencies focus their curricula on the ‘work effectively in various health care delivery settings … coordinate care … (and) work in interprofessional teams’ aspects of the SBP domain. Our POCHS curriculum, in contrast, starts by first teaching the basics of healthcare organization and delivery – culminating in how providers can effectively work within that system to impact policy and encourage reform. Until recently, published accounts of residency programs that teach health policy are few (15). We hope that our curriculum can serve as a foundation for developing new programs, as well as further refining those that do exist.

Most of our programmatic effort was in the area of knowledge and attitudes, and the overarching goal of the POCHS curriculum is to convert knowledge and awareness into practical clinical and advocacy skills. Nevertheless, there are limitations to the POCHS course and its evaluation. First, while we are hopeful that increases in knowledge may translate to better care (16, 17), we presently have no quantifiable measures of such improvements in the quality of clinical care. Second, given that the pre- and post-tests assessing SBP-related knowledge contained the same items, the tool itself may have exerted some confounding effect. Moreover, while the tests themselves demonstrated adequate internal consistencies, their validity vis-à-vis SBP remains unknown.

Despite these limitations, participant feedback suggests the course to be timely and well-received. It is obvious, however, that more GME needs to focus on SBP (18). Even though related objectives may be among the hardest to teach of all ACGME competencies (8–10), learning about current and emerging changes in healthcare is crucial to being a modern-day physician. The POCHS curriculum, while in constant development, provides a framework for teaching and assessing this competency, and can serves as a template for other residency programs looking to expand, create, or restructure their own SBP training.
